# Presenting machine learning model information to clinical end users with model facts labels

**DOI:** 10.1038/s41746-020-0253-3

**Published:** 2020-03-23

**Authors:** Mark P. Sendak, Michael Gao, Nathan Brajer, Suresh Balu

**Affiliations:** 1 Duke Institute for Health Innovation, Durham, NC USA; 2 Duke University School of Medicine, Durham, NC USA

**Keywords:** Health services, Health policy, Translational research

## Abstract

There is tremendous enthusiasm surrounding the potential for machine learning to improve medical prognosis and diagnosis. However, there are risks to translating a machine learning model into clinical care and clinical end users are often unaware of the potential harm to patients. This perspective presents the “Model Facts” label, a systematic effort to ensure that front-line clinicians actually know how, when, how not, and when not to incorporate model output into clinical decisions. The “Model Facts” label was designed for clinicians who make decisions supported by a machine learning model and its purpose is to collate relevant, actionable information in 1-page. Practitioners and regulators must work together to standardize presentation of machine learning model information to clinical end users in order to prevent harm to patients. Efforts to integrate a model into clinical practice should be accompanied by an effort to clearly communicate information about a machine learning model with a “Model Facts” label.

## Introduction

Recent advances in machine learning and artificial intelligence promise major improvements in medical diagnosis and prognosis^1^. Risk can now be estimated from a combination of pipelines of information from health records, patient reports and other sources, coupled with machine learning algorithms that produce probabilistic predictions. In the life of consumers, such algorithms underpin applications that enable the selection of routes of travel, restaurants and movies. In healthcare, however, the immediate stakes are higher, and algorithms can produce benefits and risks. Striking the right balance depends on how the algorithms are constructed and how they are used.

An interdisciplinary team including engineers, clinicians and quantitative scientists developed and validated a machine learning model to predict the risk of inpatient mortality at the time of hospital admission. The model was trained to predict the risk of death at any time during the inpatient stay. The model performed well on retrospective data, data from external hospitals, and prospectively after being integrated into the electronic health record. The team discussed workflows and agreed on the intended use of the model: to improve early alignment of goals of care, intensity of care and early engagement of palliative care for patients at high risk of inpatient mortality. During a workflow discussion, a seemingly benign question surfaced: can the model also be used to triage patients for the intensive care unit?

The potential harm to patients when using the model for a use case other than the one it was trained for was not immediately clear. Upon reflection, the 2015 experience of a team at Microsoft Research seemed pertinent. The team famously described a model developed to predict death amongst patients with pneumonia presenting to the hospital^2^. The goal was to identify which patients with pneumonia needed inpatient admission and which patients could be managed in the outpatient setting. The model found that patients with asthma were at lower risk of death, due to the fact that patients with asthma were admitted to the intensive care unit and received appropriately escalated care. If that model were integrated into clinical workflows without a clear indication for use, it’s easy to imagine patients with pneumonia complicated by asthma inappropriately treated less intensively.

The clinical utility of models is widely questioned and the need to communicate the limitations of machine learning systems has been highlighted^3,4^. However, there has not been a systematic effort to ensure that front-line clinicians actually know how, when, how not, and when not to incorporate model output into clinical decisions. Nor is there an expectation that those who develop and promote models are responsible for providing instruction of model use and for the consequences of inappropriate use.

## Standard reporting of machine learning models

In 2015, the Transparent Reporting of Multivariable Prediction Model for Individual Prognosis or Diagnosis (TRIPOD) statement was released to improve the reporting of prediction models in published literature^5^. A new initiative was recently announced to adapt the guidelines for machine learning models as well as to update clinical trial reporting for machine learning trials^6,7^. Unfortunately, models are often used without reference to the primary literature. If machine learning models are to be widely used in clinical practice, standard reporting of important model information should be coupled with use of the model, not publication of the model.

Even in published literature, model evaluations are often poorly conducted^8,9^. Model performance is often assessed using data that is easily available, rather than data that reflects the target population of actual model use. Model performance using statistical measures is often conflated with demonstrating clinical impact and utility in care delivery. Finally, clinical end users are often left ill-prepared to assess whether or not a model is generalizable to any particular setting^10,11^. Novel communication tools are needed to inform clinicians of the appropriate context and use of validated machine learning models.

Measures of model performance must also be meaningful within the context of care delivery to clinical end users. For machine learning models that discriminate between normal and abnormal states, a commonly used metric is the area under the receiver operator characteristic curve, also known as AUC^12^. AUC is a single measure of discrimination that can be interpreted as the probability of correctly ranking a randomly selected patient with the outcome as higher risk than a randomly selected patient without the outcome. The metric does not take prevalence of the outcome into account, making it difficult to interpret for rare events, and does not provide any information about calibration. Accordingly, models with improvements in AUC may be inaccurate in populations with different underlying risks or may not be anchored to appropriate absolute risk predictions. For a clinical end user receiving an alert prompted by a machine learning model, AUC is a measure that provides no actionable guidance.

## Related work

The “Model Facts” label is an example of risk communication, defined by the United States Food and Drug Administration (FDA) as “the term of art used for situations when people need good information to make sound choices”^13^. As machine learning innovations progress through different stages of diffusion, risk communication needs to be developed for different audiences and distributed via different channels^14^. Risk communication that is important during the decision stage to approve and adopt an innovation include FDA device approval summaries and medication guides as well as academic manuscripts. The “Model Facts” label specifically serves the audience of clinical end users at the implementation stage and is distributed via channels that are closely integrated with the clinical decision support.

Transparency in machine learning model reporting is not enough. As Onora O’Neill describes, “it is easy to place information in the public domain, but hard to ensure that it is in practice accessible to those for whom it might be valuable, intelligible to them if they find it, or assessable by them if they find and understand it”^15^. Ensuring that risk communication is accessible, intelligible, and assessable requires clear understanding of the objectives of the model, close collaboration with end users, and rigorous evaluation^16^. While the US FDA provides guidance on risk communication, it also acknowledges that there is no one-size-fits-all approach^13^.

Two instructive examples of risk communication research within health care are shared decision making aids and “Drug Facts” boxes. International expert consensus groups have gathered to synthesize the research and propose best practices for designing decision aids for patients^16,17^. Notable examples include https://knowyourchances.cancer.gov in the United States and https://breast.predict.nhs.uk in the United Kingdom. “Drug Facts” boxes have been rigorously evaluated in multiple randomized controlled trials^18,19^, culminating in recommendations from Congress for US FDA to consider implementing “Drug Facts” boxes^20^. Outside of health care, preliminary efforts have begun to standardize documentation to accompany a trained machine learning model^21^. There is an urgent need to design machine learning product labels that address the context-specific challenges of health care.

## The “Model Facts” label

Shortly after the experience described above, an interdisciplinary team including developers, clinicians, and regulatory experts designed the “Model Facts” label. The target audience is clinicians who make decisions supported by a machine learning model. The purpose is to collate relevant, actionable information in 1-page to ensure that front-line clinicians know how, when, how not, and when not to incorporate model output into clinical decisions. The “Model Facts” label is not meant to be comprehensive and individual sections may need to be populated over time as information about the model becomes available. For example, a model may be used in a local setting before it has been externally validated in a distinct geographical setting. There is also important information about the model, such as the demographic representation of training and evaluation data, that may need to be immediately available to an end user preceding full publication of a model.

**Fig. 1:**
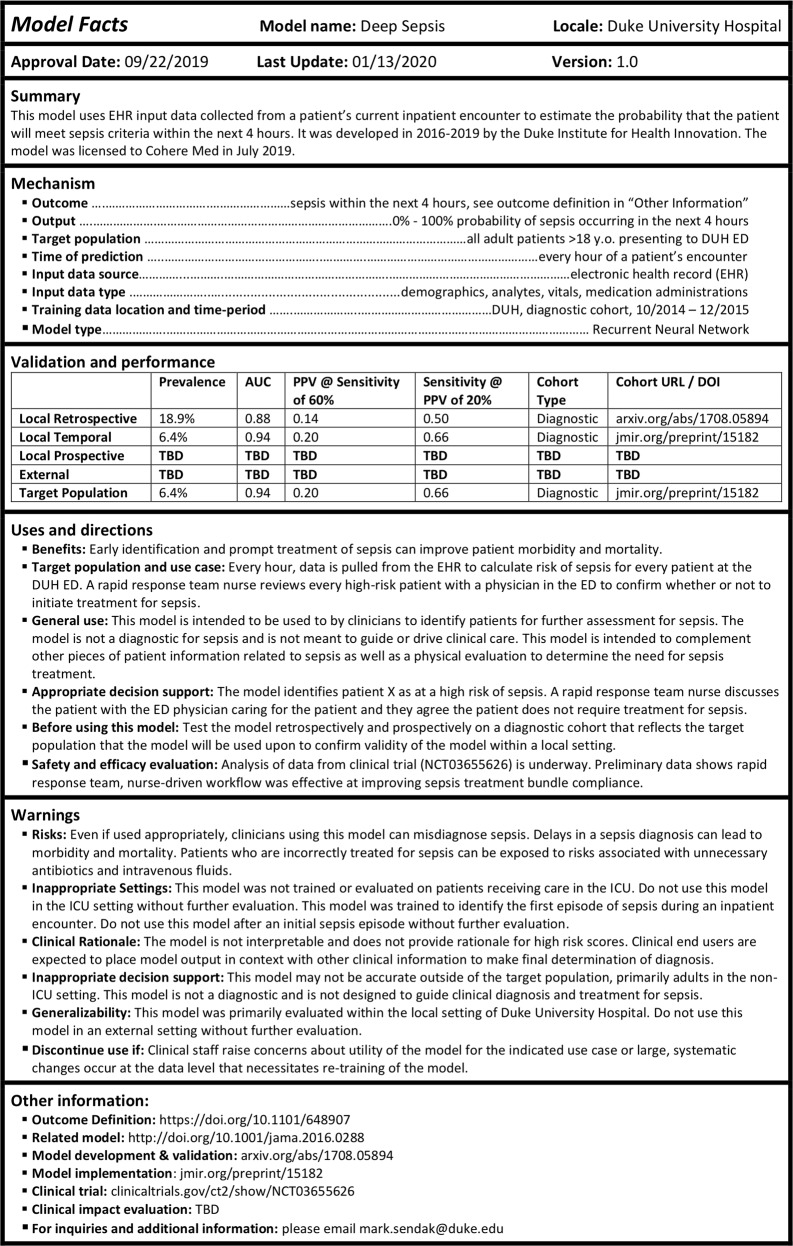
Example “Model Facts” label for a sepsis machine learning model. This “Model Facts” label provides relevant information about a sepsis prediction model to clinical end users who use the model to assist with clinical diagnosis of sepsis. AUC Area Under the ROC Curve, PPV Positive Predictive Value, DOI Digital Object Identifier, EHR Electronic Health Record, ED Emergency Department.

Figure 1 illustrates an example “Model Facts” label designed for a sepsis prediction model. The major sections of the “Model Facts” label include the model name, locale, and version, summary of the model, mechanism of risk score calculation, validation and performance, uses and directions, warnings, and other information. The structure is meant to mirror product information for food, drugs and devices. Publication hyperlinks in the “Validation and performance” and “Other information” section point to additional details.

Two sections of the “Model Facts” label that are rarely discussed in machine learning model publications are “Uses and directions” and “Warnings”. Every machine learning model is trained for a specific task and the boundary lines around that task must be clearly communicated. In our example, warnings are provided to only use the model within settings in which the model was evaluated, to not use the model after a patient develops a first episode of sepsis, and to not use the model in an intensive care unit without further evaluation. There is also a warning against automated treatment assignment.

“Model Facts” labels need to be localized and need to be updated over time. Similar to how antimicrobial sensitivity data guide use of antibiotics within a local population, “Model Facts” labels include information about model performance within the local population. If a model is adopted in a new setting, a new “Model Facts” label needs to be generated and distributed to clinical end users. The target population of model use is also specified in both the “Uses and directions” and “Validation and performance” sections. The version of the “Model Facts” label is documented and version control with documentation of changes should be accessible to all end users^22^. Use of the model and the “Model Facts” label also needs to be approved by governance structures that function similarly to pharmacy and therapeutics committees that monitor use of medications and adverse outcomes.

The structure of our “Model Facts” label presented in Fig. 1 requires rigorous testing and evaluation. It is not meant to be immediately adopted, but to spark dialogue and to be iterated upon and critiqued by a broad group of stakeholders. Risk communication research advises against only using words in communication material^16^ and we hope that other teams implementing machine learning tools create their own versions of “Model Facts” labels.

Many questions remain about the design of the “Model Facts” label and how to make this information accessible, intelligible, and assessable to clinicians. Should the information be accessible within the electronic health record, software applications, an online registry, or some combination? And how is information presented to an end user when it’s not immediately clear that a model was involved, for example with a text notification? Despite unanswered questions, without bringing together practitioners and regulators to standardize presentation of machine learning model information to clinical end users, we risk significant harm to patients. Any effort to integrate a model into clinical practice should be accompanied by an effort to clearly communicate how, when, how not, and when not to incorporate model output into clinical decisions.
